# Side-effects in women treated with adjuvant endocrine therapy for breast cancer

**DOI:** 10.1016/j.breast.2025.104416

**Published:** 2025-02-11

**Authors:** L.M. Rademaker, R. Gal, A.M. May, M.C.T. Batenburg, F. van der Leij, R.M. Bijlsma, H.M. Verkooijen, A. Doeksen, M.F. Ernst, D.J. Evers, C.C. van der Pol, E.M. Monninkhof

**Affiliations:** a Julius Center for Health Sciences and Primary Care, University Medical Center Utrecht, Utrecht University, Utrecht, the Netherlands; b Division of Imaging and Oncology, University Medical Center Utrecht, Utrecht University, Utrecht, the Netherlands; c Department of Radiation Oncology, University Medical Center Utrecht, Utrecht, the Netherlands; d Department of Medical Oncology, University Medical Centre Utrecht, Cancer Centre, Utrecht, the Netherlands; e Department of Surgery, St. Antonius Hospital, Nieuwegein, the Netherlands; f Department of Surgery, Alexander Monro Clinics, Bilthoven, the Netherlands; g Department of Surgery, Hospital Group Twente, Almelo, the Netherlands; h Department of Surgery, Alrijne Hospital, Leiderdorp, the Netherlands

**Keywords:** Adjuvant therapy, Endocrine therapy, ER positive, Side-effects, Discontinuation of therapy

## Abstract

Many women with breast cancer prematurely discontinue adjuvant endocrine therapy, leading to increased mortality. We performed a cross-sectional survey (n = 456) within the Dutch UMBRELLA-cohort to gain insight into the prevalence of side-effects and its association with premature discontinuation. Almost all current endocrine therapy users experienced side-effects (92.7 %), most frequent were vasomotor- and musculoskeletal symptoms. The most reported reason for premature discontinuation was side-effects (88.1 %). Former treatment with chemotherapy was associated with more reported side-effects (97.2 % vs 82.5 %, ORadj 6.31), but less premature discontinuation of endocrine therapy (18.6 % vs 28.6 %, p-value 0.016).

## Highlights

• Cross-sectional survey within the UMBRELLA breast cancer cohort, response rate 77.6 %.
• 93 % of current endocrine therapy users experienced side-effects.
• Most reported side-effects were musculoskeletal- and vasomotor symptoms.
• The most reported reason for stopping prematurely was side-effects (88.1 %).
• Chemotherapy is associated with more side-effects and less premature discontinuation.

## 1 Introduction

Endocrine therapy, such as tamoxifen and aromatase inhibitors, significantly decreases recurrence and mortality rates in women with hormone-sensitive breast cancer [[1], [2], [3]]. The prescribed duration of endocrine therapy varies from five to ten years, which is often not completed [[4], [5], [6]]. Treatment-related side-effects can cause premature discontinuation [6]. These side-effects might be the result of changes in hormone balance, causing menopausal symptoms [7]. The patients’ abilities to cope with side-effects also influence discontinuation rates [8]. Many patients who are prescribed endocrine therapy also receive chemotherapy, which can also lead to often long-lasting side-effects [9,10].

To reduce premature discontinuation of endocrine therapy, it is important to 1) determine the prevalence and type of side-effects, 2) determine the prevalence and reasons for premature discontinuation and 3) identify whether chemotherapy is associated with side-effects and/or discontinuation of endocrine therapy.

## 2 Methods

### 2.1 Study design

This cross-sectional survey study was performed within the Utrecht cohort for Multiple BREast cancer intervention studies and Long-term evaLuAtion (UMBRELLA) including patients with breast cancer referred to the University Medical Center Utrecht for radiotherapy [11]. Patients were asked consent for collection of clinical data and patient-reported outcomes. Women with an indication for endocrine therapy who actively participated in UMBRELLA during July 2020 were sent a self-designed survey (Fig. S1). The survey consisted of five multiple-choice questions concerning endocrine therapy use, side-effects, and, if applicable, reasons for discontinuation, all questions included an option to give another answer than prespecified.

Age and education were obtained from UMBRELLA [11]. Clinical data were obtained from the Netherlands Cancer Registry [12]. When menopausal state was unknown, women ≥55 years of age were considered post-menopausal and menopausal status of women <55 years was reported as missing.

### 2.2 Statistical analysis

Descriptive statistics were used for demographic and clinical characteristics, stratified by treatment with chemotherapy. Categorical variables were expressed as counts and proportions, continuous variables as mean ± standard deviation (SD).

Baseline differences between participants treated with or without chemotherapy were tested using Χ^2^-square for categorical variables and the independent *t*-test for continuous variables. Logistic regression analysis was performed to examine the associations between treatment with chemotherapy and side-effects. Logistic regression analysis was adjusted for age, time since diagnosis, targeted therapy, and education level, and reported as odds ratios (OR) and 95 % confidence intervals (CI). Analyses were performed using SPSS (version 26).

## 3 Results

Of the 3238 UMBRELLA participants, 588 potentially eligible women received a survey whereof 456 women (77.6 %) completed the survey and were actually prescribed endocrine treatment (Fig. S2). Mean age of included women was 60.8 years (SD 10.1; Table 1). The mean time between diagnosis and survey was 4.6 years (SD 1.2, range 2.5–7.3). Most tumors were moderately differentiated (54.4 %) and HER2/neu negative (86.4 %). Participants who received chemotherapy were significantly younger than participants who did not receive chemotherapy (58.1 vs 67.9 years, p < 0.001).

**Table 1:** Characteristics of participants who completed the endocrine therapy survey stratified for chemotherapy (yes/no).

	Total	No chemotherapy	Chemotherapy	Between group differences (p-value)
**Total, N (%)**	456 (100.0)	161 (35.3)	295 (64.7)	
**Current endocrine therapy user, N (%)**^a^				0.016
Yes	313 (68.6)	97 (60.2)	216 (73.2)	
No, stopped prematurely	101 (22.1)	46 (28.6)	55 (18.6)	
No, treatment time completed	42 (9.2)	18 (11.2)	24 (8.1)	
**Age in years at survey, mean ± SD**	60.8 ± 10.1	67.1 ± 9.5	57.4 ± 8.7	<0.001
**Education, N (%)**				0.027
Primary or lower secondary school (VMBO)	67 (14.7)	31 (19.3)	36 (12.2)	
Higher secondary school (HAVO/VWO) or post-secondary vocational education (MBO)	107 (23.5)	32 (19.9)	75 (25.4)	
College or university	189 (41.4)	58 (36.0)	131 (44.4)	
Missing/unknown	93 (20.4)	40 (24.8)	53 (18.0)	
**Time in years between diagnosis and survey**				
**Mean ± SD**	4.6 ± 1.2	4.3 ± 1.2	4.7 ± 1.2	0.003
**Range**	2.5–7.3	2.5–7.1	2.5–7.3	
**Time in years between end of chemotherapy and survey**				
**Mean ± SD**			4.2 ± 1.2	
**Range**			1.2–6.7	
**Menopausal state at diagnosis, N (%)**^a^				<0.001
Pre- and perimenopausal	180 (39.5)	32 (19.9)	148 (50.2)	
Postmenopausal	251 (55.0)	124 (77.0)	127 (43.1)	
Unknown	25 (5.5)	5 (3.1)	20 (6.8)	
**Breast cancer grading, N (%)**				<0.001
Well differentiated	68 (14.9)	33 (20.5)	35 (11.9)	
Moderately differentiated	248 (54.4)	111 (68.9)	137 (46.4)	
Poorly differentiated	89 (19.5)	14 (8.7)	75 (25.4)	
Unknown/not measured	51 (11.2)	3 (1.9)	48 (16.3)	
**TNM, N (%)**^a^				<0.001
Stage I	136 (29.8)	75 (46.6)	61 (20.7)	
Stage IIA	181 (39.7)	61 (37.9)	120 (40.7)	
Stage IIB	73 (16.0)	16 (9.9)	57 (19.3)	
Stage IIIA, IIIB and IIIC	43 (9.4)	4 (2.5)	39 (13.2)	
Unknown/missing	23 (5.0)	5 (3.1)	18 (6.1)	
**HER2/neu status, N (%)**^a^				<0.001
Negative	394 (86.4)	157 (97.5)	237 (80.3)	
Positive	59 (12.9)	2 (1.2)	57 (19.3)	
Unknown/not measured	3 (0.6)	2 (1.2)	1 (0.3)	
**Treatment besides endocrine therapy, N (%)**^b^				
Radiotherapy, yes	454 (99.6)	159 (98.8)	298 (100.0)	0.055
Targeted therapy, yes	59 (12.9)	0 (0.0)	59 (20.0)	<0.001

Abbreviations: **HER2/neu** Human Epidermal growth factor Receptor 2, **SD** Standard Deviation, **TNM** Tumor Node Metastasis (Classification of Malignant Tumors).

a Due to rounding up or down to one decimal, the total of percentages can deviate 0.1 % from adding up to 100.0 %.

b Subcategories are not the sum of main categories, because participants could have received more than one treatment.

From the 456 participants, 313 (68.6 %) were currently using endocrine therapy and 101 (22.1 %) stopped prematurely (Table 2). Of the current users, 92.7 % experienced side-effects. The most reported side-effects were vasomotor symptoms (75.1 %) and joint and/or muscle symptoms (69.3 %).

**Table 2:** Side effects in participants currently using endocrine therapy, stratified by former treatment with chemotherapy.

	Total	No chemotherapy	Chemotherapy	OR (95%CI)	OR_adj_ (95%CI)
**Total currently using endocrine therapy, N**	313	97	216		
**Type of endocrine therapy, N (%)**					
Tamoxifen	122 (39.0)	41 (42.3)	81 (37.5)		
Aromatase inhibitor	171 (54.6)	52 (53.6)	119 (55.1)		
LHRH agonist	8 (2.6)	2 (2.1)	6 (2.8)		
Unclear/other	12 (3.8)	2 (2.1)	10 (4.6)		
**Current endocrine therapy users experiencing side-effects, N (%)**	290 (92.7)	80 (82.5)	210 (97.2)	7.44 (2.83–19.54)	6.31 (1.75–22.84)
**Type of side-effects, N (%)**^b^					
Vasomotor symptoms	218 (75.1)	58 (72.5)	160 (76.1)	1.21 (0.68–2.18)	0.93 (0.44–1.98)
Joint and/or muscle symptoms	201 (69.3)	50 (62.5)	151 (71.9)	1.54 (0.89–2.64)	1.07 (0.53–2.18)
Cognitive problems	135 (46.6)	26 (32.5)	109 (51.9)	2.24 (1.31–3.85)	2.36 (1.20–4.63)
Sleeping problems	119 (41.0)	29 (36.3)	90 (42.9)	1.32 (0.78–2.24)	1.24 (0.63–2.44)
Emotional complaints^b^	113 (39.0)	32 (40.0)	81 (38.6)	0.94 (0.56–1.60)	0.91 (0.47–1.78)
Emotional fluctuations	95 (32.8)	25 (31.3)	70 (33.3)		
Depressive symptoms	33 (11.4)	7 (8.8)	26 (12.4)		
Anxiety problems	13 (4.5)	3 (3.8)	10 (4.8)		
Panic attacks	9 (3.1)	4 (5.0)	5 (2.4)		
Loss of libido	108 (37.2)	24 (30.0)	84 (40.0)	1.56 (0.90–2.70)	0.97 (0.47–1.99)
Other side-effects^c^	105 (32.0)	26 (32.5)	79 (37.6)	1.25 (0.73–2.16)	1.02 (0.52–2.01)
**Number of side-effects, N (%)**^a^					
0	23 (7.3)	17 (17.5)	6 (2.8)		
1–2	96 (30.7)	34 (35.1)	62 (28.7)		
3–4	117 (37.4)	33 (34.0)	84 (38.9)		
5–8	76 (24.3)	13 (13.4)	63 (29.2)		
Unknown (not specified which side-effects)	1 (0.3)	0	1 (0.5)		

Abbreviations: **OR** odds ratio, **OR**_**adj**_ odds ratio adjusted for age, time between diagnosis and survey, former treatment with targeted therapy and education, **CI** confidence interval, **LHRH** luteinizing hormone-releasing hormone.

a Due to rounding up or down to one decimal, the total of percentages can deviate 0.1 % from adding up to 100.0 %.

b Subcategories are not the sum of main categories, because participants could have multiple answers.

c Other side effects reported: changes in weight, tiredness, hair loss, bowel problems, blurry sight, skin problems, dry mouth, dry eyes, condition loss, fluid retention, unwanted hair growth, more rapid aging, chest pain and bone problems.

Endocrine therapy users who have been treated with chemotherapy experienced more side-effects (97.2 % vs 82.5 %, OR_adj_ 6.35, 95 % CI 1.76–22.96) compared to participants who did not receive chemotherapy. They reported more cognitive problems compared to participants who did not receive chemotherapy (51.9 % vs 32.5 %, OR_adj_ 2.36, 95%CI 1.20–4.63). Participants with past chemotherapy and premature discontinuation of endocrine therapy reported less vasomotor symptoms as side-effects compared to the non-chemotherapy group (45.3 % vs 77.8 %; OR_adj_ 0.16, 95%CI 0.04–0.57). Participants who did not receive chemotherapy more often stopped prematurely than participants that received chemotherapy (28.6 % vs 18.6 %; Table 1).

In total the most reported reason for stopping prematurely was side-effects (88.1 %; Table 3). The most reported side-effects that induced discontinuation were joint and/or muscle symptoms (74.2 %) and vasomotor symptoms (58.4 %).

**Table 3:** Side effects and reasons to stop in participants who discontinued endocrine therapy prematurely, stratified by former treatment with chemotherapy.

	Total	No chemotherapy	Chemotherapy	OR (95%CI)	OR_adj_ (95%CI)
**Total prematurely stopped using endocrine therapy, N**	101	46	55		
**Type of endocrine therapy before stop, N (%)**^b^					
Tamoxifen	69 (68.3)	26 (56.5)	43 (78.2)		
Aromatase inhibitor	54 (53.5)	27 (58.7)	27 (49.1)		
LHRH agonist	4 (4.0)	0	4 (7.3)		
Other	16 (15.8)	11 (23.9)	5 (9.1)		
**Reasons to prematurely stop endocrine therapy, N (%)**^b^					
Side-effects	89 (88.1)	36 (78.3)	53 (96.4)	2.47 (0.96–6.35)	1.99 (0.55–7.20)
Higher risk for endometrial cancer	7 (6.9)	1 (2.2)	6 (10.9)		
Pregnancy wish	4 (4.0)	0	4 (7.3)		
Don't want to take any medicine	10 (9.9)	3 (6.5)	7 (12.7)		
Other reasons, such as small expected survival benefit, impaired QoL, recommended by the physician	34 (33.7)	16 (34.8)	18 (32.7)		
**Patients who stopped taking endocrine therapy prematurely because of side-effects, N**	89	36	53		
**Type of side-effects that caused stop, N (%)**^b^					
Joint and/or muscle symptoms	66 (74.2)	25 (69.4)	41 (77.4)	1.50 (0.58–3.92)	0.84 (0.24–3.02)
Vasomotor symptoms	52 (58.4)	28 (77.8)	24 (45.3)	0.24 (0.09–0.61)	0.16 (0.04–0.57)
Emotional complaints^b^	45 (50.6)	18 (50.0)	27 (50.9)	1.04 (0.45–2.42)	0.93 (0.31–2.79)
Emotional fluctuations	24 (27.0)	9 (25.0)	15 (28.3)		
Depressive symptoms	20 (22.5)	7 (19.4)	13 (24.5)		
Anxiety problems	6 (6.7)	2 (5.6)	4 (7.5)		
Panic attacks	6 (6.7)	1 (2.8)	5 (9.4)		
Sleeping problems	39 (43.8)	19 (52.8)	20 (37.7)	0.54 (0.23–1.28)	0.37 (0.12–1.14)
Cognitive problems	38 (42.7)	12 (33.3)	26 (52.4)	1.93 (0.80–4.63)	0.83 (0.27–2.56)
Loss of libido	33 (37.1)	9 (25.0)	24 (49.0)	2.48 (0.98–6.28)	1.44 (0.46–4.68)
Other side-effects^c^	29 (32.6)	9 (25.0)	20 (37.7)	1.82 (0.71–4.64)	3.27 (0.87–12.31)
**Number of side-effects that caused stop, N (%)**^a^					
1–2	33 (37.1)	14 (38.9)	19 (35.8)		
3–4	27 (30.3)	10 (27.8)	17 (32.1)		
5–7	29 (32.6)	12 (33.3)	17 (32.1)		

Abbreviations: **OR** odds ratio, **OR**_**adj**_ odds ratio adjusted for age, time between diagnosis and survey, former treatment with targeted therapy and education, **CI** confidence interval, **LHRH** luteinizing hormone-releasing hormone, **QoL** quality of life.

a Due to rounding up or down to one decimal, the total of percentages can deviate 0.1 % from adding up to 100.0 %.

b Subcategories are not the sum of main categories, because participants could have multiple answers and received more than one treatment.

c Other side effects reported: changes in weight, tiredness, fluid retention, bone problems, hair loss, itch, reduced QoL.

## 4 Discussion

In this Dutch breast cancer cohort, almost all users of endocrine therapy experienced side-effects. This is in line with earlier studies, also reporting a very high prevalence of side-effects, predominantly vasomotor and musculoskeletal symptoms [[13], [14], [15], [16], [17]].

Consistent with the literature, one in four participants stopped prematurely with endocrine therapy, mostly because of side-effects [[17], [18], [19]]. Due to our cross-sectional design, it is possible that respondents who were currently using endocrine therapy stop prematurely after survey completion. Therefore, the actual number of women who prematurely stopped may be higher in clinical practice.

Participants who were exposed to chemotherapy reported side-effects more often. These participants may also experience (long-term) consequences of chemotherapy [9,10]. In this study, we cannot distinguish between causes of side-effects.

The prevalence of participants that prematurely stopped endocrine therapy was lower in the chemotherapy group than in the non-chemotherapy group. Since prognosis is worse in the chemotherapy group, for these women the beneficial effects of endocrine therapy might outweigh the side-effects.

The prospective UMBRELLA cohort, consisting of pre-, peri- and postmenopausal women, provided real-world insight on the prevalence of side-effects, which is a strength of this study [11]. Our survey revealed a complete overview of the experienced side-effects. Furthermore, we asked the patients themselves what their reason for discontinuation was and if applicable which side-effect. There are more influencing factors for premature discontinuation than just the presence of side-effects. In contrast, other studies focused on the association between side-effects and premature discontinuation [8]. The response rate of 77.6 % in our survey was high, which indicates the importance of this subject. However, response might still be selective and side-effects could be slightly overreported. Another study limitation may be the use of a self-designed, not validated, survey.

Based on the high prevalence of side-effects in patients with breast cancer receiving endocrine therapy, it is important to counsel patients well prior to use of endocrine therapy. Self-efficacy and decisional balance are potential modifiable factors [8]. Physical interventions, pharmaceutical therapy and cognitive behavioral therapy are promising for prevention, treatment or coping with side-effects [15,20]. In conclusion, this research adds real-world insight to the evidence that side-effects are highly prevalent and might induce premature discontinuation of endocrine treatment.

## CRediT authorship contribution statement

**L.M. Rademaker:** Writing – review & editing, Writing – original draft, Methodology, Formal analysis, Data curation, Conceptualization. **R. Gal:** Writing – review & editing, Supervision, Methodology. **A.M. May:** Writing – review & editing. **M.C.T. Batenburg:** Writing – review & editing. **F. van der Leij:** Writing – review & editing. **R.M. Bijlsma:** Writing – review & editing. **H.M. Verkooijen:** Writing – review & editing. **A. Doeksen:** Data curation. **M.F. Ernst:** Data curation. **D.J. Evers:** Data curation. **C.C. van der Pol:** Data curation. **E.M. Monninkhof:** Writing – review & editing, Supervision, Methodology, Conceptualization.

## Funding

This research did not receive any specific grant from funding agencies in the public, commercial or not-for-profit sectors.

## Declaration of competing interest

None declared.
