# Supplementary material for: Side-effects in women treated with adjuvant endocrine therapy for breast cancer
Source: Breast. 2025 Feb 11;80:104416. doi: 10.1016/j.breast.2025.104416 (PMC11880597; doi:10.1016/j.breast.2025.104416)
Supplement: Multimedia component 1 [file mmc1.docx]

**Figure S1. Survey UMBRELLA Endocrine therapy**

1. According to our information, you have been prescribed endocrine therapy. Are you using it now?
   - Yes -> *go to question 2 and 3*
   - This was prescribed, but I am not using it (anymore) -> *go to question 4 and 5*
   - No, I was not prescribed endocrine therapy -> *end of survey*
2. Which endocrine therapy are you using now? (multiple answers are possible)

- Tamoxifen
- Aromatase inhibitor
- LHRH agonist
- Other, namely_________________________________

1. If yes, are you experiencing side-effects? (you can tick multiple boxes)
   - Yes, namely:
     - Hot flashes
     - Night sweats
     - Concentration and/or memory problems
     - Sleeping problems
     - Joint and/or muscle pain
     - Loss of libido
     - Emotional complaints, if yes:
       - Anxiety
       - Panic attacks
       - Depressive complaints
       - Emotional fluctuations
     - Other side-effect, namely_____________________
   - No
2. If no, for how long did you use endocrine therapy?

| Medicine | Start date | Stop date | Prescribed duration |
| --- | --- | --- | --- |
|  |  |  |  |
|  |  |  |  |
|  |  |  |  |

1. What was the reason for stopping with endocrine therapy? (you can tick multiple boxes)
   - Completed treatment time
   - Side-effects, namely:
     - Hot flashes
     - Night sweats
     - Concentration and/or memory problems
     - Sleeping problems
     - Joint and/or muscle pain
     - Loss of libido
     - Emotional complaints, if yes:
       - Anxiety
       - Panic attacks
       - Depressive complaints
       - Emotional fluctuations
     - Other side-effect, namely_____________________
   - I want to get pregnant.
   - I do not want to take medicine anymore
   - Higher risk for endometrial cancer
   - Other reason, namely:

________________________________________________
