# Supplementary figures and images for: Side-effects in women treated with adjuvant endocrine therapy for breast cancer
Source: Breast. 2025 Feb 11;80:104416. doi: 10.1016/j.breast.2025.104416 (PMC11880597; doi:10.1016/j.breast.2025.104416)

**Figure S2. Flowchart**


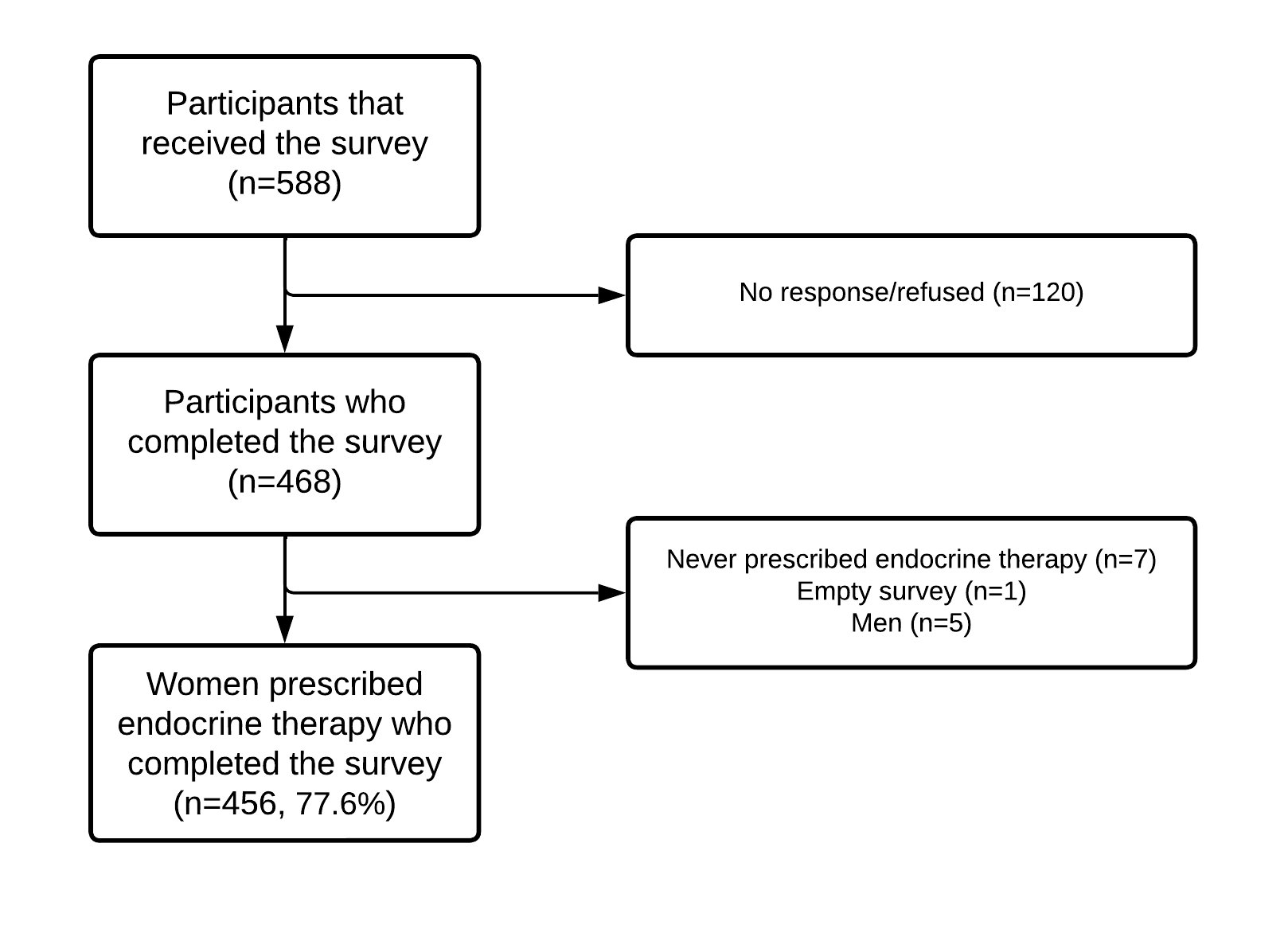

Supplement: Multimedia component 2 [file mmc2.docx]
